# Rechallenge of ruxolitinib after momelotinib-related toxicity in a 74-yo woman with secondary myelofibrosis: a case report

**DOI:** 10.3389/fmed.2026.1878696

**Published:** 2026-07-01

**Authors:** Jose Miguel Torregrosa Diaz, Laura Cailly, Maria Pilar Gallego Hernanz, Hélène Gardeney, Emilie Cayssials

**Affiliations:** 1 Service d'Hématologie Oncologique et Thérapie Cellulaire, Poitiers' University Hospital, INSERM CIC 1402, Poitiers, France; 2 Service d'Hématologie Oncologique et Thérapie Cellulaire, Poitiers' University Hospital, University of Poitiers, INSERM CIC 1402, Poitiers, France; 3 Service d'Hématologie Oncologique et Thérapie Cellulaire, Poitiers' University Hospital, University of Poitiers, INSERM CIC 1402, Poitiers, France

**Keywords:** drug-induced adverse effects, momelotinib, peripheral neuropathy, ruxolitinib, secondary myelofibrosis, switch-back

## Abstract

**Background:**

In the era of new JAK inhibitors (JAKi), combination therapy, and other targeted therapies, it seems especially important to optimize the treatment options. The rechallenge of a JAKi becomes one of the possibilities, already reported in the literature, the better documented, between ruxolitinib and fedratinib. To date, no case of rechallenge between ruxolitinib and momelotinib (MMB) has been reported.

**Short report:**

We present a case of secondary-to-ET myelofibrosis in a 74-year-old female initially treated by high-dose ruxolitinib, switched to momelotinib because of transfusion-dependent anemia, and who developed significant peripheral neuropathy and arthralgias during treatment with momelotinib, unsuccessfully managed by dose reductions, and subsequently re-switched to low-dose ruxolitinib due to the improvement of anemia after MMB and the objective of maintaining symptom control.

**Conclusion:**

This case highlights the possibility of revisiting JAKi after momelotinib, depending on therapeutic objectives and to manage adverse effects.

## Introduction

Myelofibrosis (MF) is a chronic myeloproliferative neoplasm characterized by clonal myeloid proliferation, progressive bone marrow fibrosis, cytopenias, splenomegaly, and for most of patients, a heavy burden of constitutional symptoms that can heavily impair the quality of life of patients. Janus kinase (JAK) 1/2 inhibition with ruxolitinib remains the standard of care for symptomatic primary and secondary MF, based on the COMFORT I and II trials, which demonstrated durable reductions in spleen volume and symptom improvement, but limited impact on disease-modifying endpoints and anemia (1–4). Anemia and transfusion dependence are frequent in MF and strongly affect quality of life and prognosis, yet they are often exacerbated by ruxolitinib, creating a clinical need for alternative strategies in patients with significant baseline cytopenias or in the presence of toxicities.

Momelotinib is an oral JAK1/2 and activin A receptor type I (ACVR1) inhibitor that has shown activity across the three major clinical hallmarks of MF: anemia, splenomegaly, and constitutional symptoms. Through ACVR1 inhibition and hepcidin suppression, momelotinib has demonstrated the ability to improve MF-related anemia and reduce transfusion requirements, including in patients previously treated with ruxolitinib. Integrated analyses of phase 3 trials and emerging real-world cohorts have confirmed meaningful anemia responses and acceptable overall tolerability (5, 6). However, they have also highlighted treatment-emergent adverse events such as thrombocytopenia, gastrointestinal toxicity, and peripheral neuropathy in a subset of patients (7).

The sequential use and rechallenge of JAK inhibitors are increasingly being explored as part of the pharmacopeia for MF as the therapeutic armamentarium for MF expands. Ruxolitinib rechallenge after prior intolerance or resistance, and switching between ruxolitinib and fedratinib, have been reported as feasible approaches in selected patients, potentially restoring symptom control or hematologic benefit (8). However, data on sequencing strategies involving momelotinib remain sparse, and, to our knowledge, no case has yet been published describing a ruxolitinib rechallenge after momelotinib-related toxicity, in this case, a peripheral neuropathy.

Here we report the case of a 74-year-old woman with secondary to essential thrombocythemia MF who was switched from high-dose ruxolitinib to momelotinib for transfusion-dependent anemia, achieved a marked and sustained hematologic response, but developed dose-limiting peripheral neuropathy and arthralgias. We describe the subsequent successful reintroduction of low-dose ruxolitinib, with resolution of neuropathy and maintenance of anemia control, and discuss the implications of this experience for individualized JAK inhibitor sequencing and toxicity management in MF.

## Case description

A 74-year-old female patient with a significant medical history was referred to our institution for hematologic consultation. Her cardiovascular history included a transient ischemic attack in 2000 with right hemiparesis that resolved completely without sequelae, left carotid stenosis at 30%, dyslipidemia, and cutaneous malignancy of the right ear treated with surgical excision in 2012. Current medications included nebivolol 5 mg daily, amlodipine 10 mg daily, levothyroxine 125 μg daily, and acetylsalicylic acid 75 mg daily. Baseline renal function showed a GFR of 47 mL/min (CKD stage 3b), with no other significant comorbidities.

The patient was diagnosed with essential thrombocythemia (ET) in 2010. NGS identified two pathogenic variants in *MPL* (c.1514_1515 delinsAT, VAF 46%; and c.1495 C>T, VAF 45%) and one variant in TET2 (p.H1716lfs^*^3, VAF 28%).

Initial treatment consisted of hydroxyurea at standard doses for 3 years, which was discontinued due to a drug-related toxicity: lower extremity ulcers. The patient was subsequently switched to anagrelide. In early 2019, after three months of anagrelide, it was discontinued due to progressive cytopenias with hemoglobin between 90–100 g/L and platelets between 80 and 90 × 10^?^/L.

In February 2020, a year after anagrelide cessation, the patient presented with clinical and laboratory features consistent with secondary myelofibrosis. At this time, her hemoglobin was 83 g/L, white blood cell count 7.83 × 10^?^/L with 6% erythromyelemia, platelets 70 × 10^?^/L, and LDH elevated at three times the upper limit of normal. Constitutional symptoms included significant articular and bone pain requiring oral morphine, weight loss of 3 kg over 6 weeks with no ongoing diet, and anorexia. Physical examination documented non-tender splenomegaly with 5 cm splenic edge below the costal margin. Bone marrow biopsy revealed grade 2 myelofibrosis (MF-2). Given this presentation, the patient was initiated on ruxolitinib at 10 mg twice daily.

In the first quarter of 2022, the patient transferred care to our institution at University Hospital of Poitiers while continuing ruxolitinib 10 mg twice daily. Clinical assessment showed regression of splenomegaly to 4 cm splenic edge, complete resolution of constitutional symptoms, though persistent asthenia (which was attributed to anemia). However, hemoglobin progressively declined, and the patient became transfusion-dependent 3 months after her first visit to our clinic, requiring red cell transfusion approximately every 6 weeks.

Despite this anemia-related burden, the ruxolitinib dose was escalated to 15 mg twice daily to achieve splenomegaly response, and erythropoietin (40,000 IU weekly) was added. This combination stabilized her hemoglobin levels, with splenomegaly progressively resolving and becoming impalpable. However, persistent transfusion dependence was noted, occurring approximately every 8 weeks. This had a particular negative impact on this patient's living disease, as even at this relatively low frequency of visits to the hospital, she felt handicapped for her daily life because of the visits and the asthenia. Laboratory investigation revealed iron overload, with ferritin levels of 1,200 ng/mL and transferrin saturation of 95%, necessitating iron chelation therapy. The patient reported a marked difficulty with disease burden despite symptomatic improvement.

Given the inadequate anemia response to ruxolitinib and erythropoietin, and the emerging data suggesting a potential benefit of momelotinib in myelofibrosis-associated anemia, she was enrolled in a compassionate use program to receive momelotinib 200 mg daily in late March 2023, with no EPO support. At the initiation of momelotinib, she had no palpable splenomegaly, no constitutional symptoms, and no evidence of extramedullary disease.

The hematologic response to momelotinib was remarkable. Within the first 2 months of therapy, hemoglobin rose from 76 to 102 g/L, achieving transfusion independency, then subsequently to 136 g/L over several months (maximum hemoglobin level during momelotinib treatment). The patient reported reduced asthenia. However, approximately 3 months after initiation of momelotinib, the patient began experiencing progressive arthralgias and distal paresthesia. These symptoms became clinically significant during the 6-month evaluation on MMB. She experienced an important limitation in fine hand activities and a slight one in walking.

Physical and neurologic examination supported a peripheral neuropathy. Electromyography and nerve conduction studies (ENMG) confirmed a length-dependent sensory axonal neuropathy with distal predominance. The clinical and electrophysiologic findings, along with the temporal relationship to momelotinib initiation, were consistent with drug-induced peripheral neuropathy.

Given the severity of symptoms and their impact on quality of life, the momelotinib dose was sequentially reduced to 150 mg daily, then to 100 mg daily. While this dose reduction resulted in partial improvement of neuropathy symptoms, resolution was incomplete, and the patient remained symptomatic.

Given the persistent neuropathy despite dose reduction, despite momelotinib's significant contribution to her hemoglobin response, a decision was made to discontinue momelotinib and re-initiate ruxolitinib at a lower dose (5 mg twice daily), still without erythropoietin, as an improvement in spleen volume was not necessary at this moment, but to maintain symptoms and splenomegaly response.

**Figure 1:**
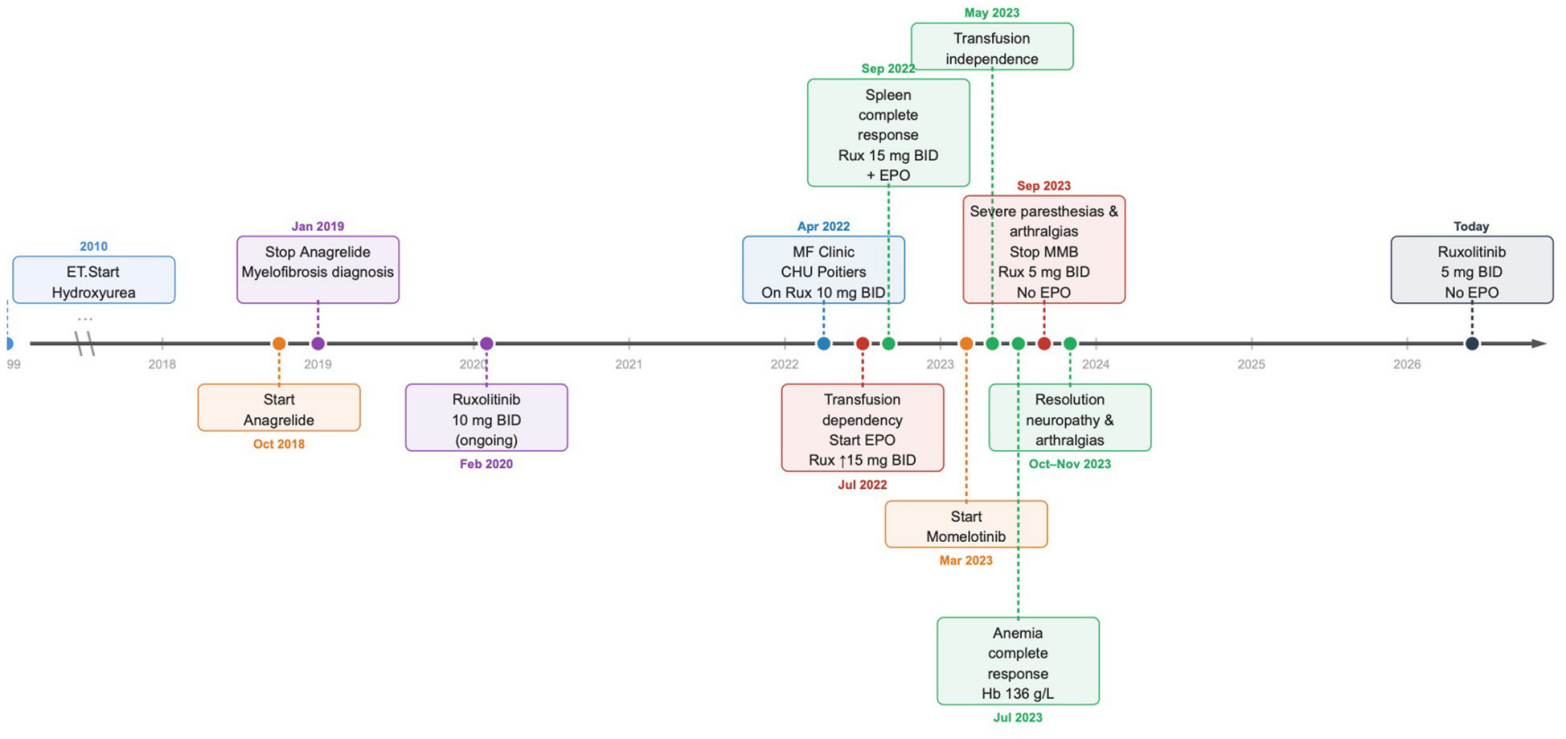
shows the timeline of different treatments and responses.

Following the switch to low-dose ruxolitinib (5 mg twice daily), the patient experienced a complete resolution of peripheral neuropathy symptoms and arthralgias in the following 6 to 7 weeks. Importantly, hemoglobin remained above 120 g/L with fluctuations (range 120–136 g/L), demonstrating sustained hematologic control despite the substantial dose reduction from ruxolitinib 15 mg twice daily plus erythropoietin to 5 mg twice daily monotherapy. Splenomegaly remained absent on physical examination, and constitutional symptoms did not recur. The patient currently remains on low-dose ruxolitinib monotherapy with stable disease and improved quality of life. Figure 1 shows the timeline of events.

## Discussion

This case illustrates the complex therapeutic journey of a patient with ET-secondary myelofibrosis treated sequentially with JAK inhibitors, ultimately complicated by drug-induced peripheral neuropathy during momelotinib escalation and rechallenge of ruxolitinib at a lower dose with a favorable outcome.

Ruxolitinib remains the gold-standard JAK inhibitor for symptomatic myelofibrosis based on the COMFORT trials and subsequent clinical experience (3, 4). However, its limitations in addressing myelofibrosis-associated anemia have motivated investigation of alternative JAK inhibitors and combination strategies. Momelotinib, through its dual inhibition of JAK1/2 and ACVR1, provides enhanced benefit for anemia, and clinical trial data suggest a superior anemia response compared to ruxolitinib (6, 9). However, accumulating real-world experience raises concerns regarding momelotinib tolerability profile, particularly regarding peripheral neuropathy (5, 10, 11).

Other series have reported the rechallenge of ruxolitinib from fedratinib and vice versa (8). Our case documents a patient who achieved an exceptional anemia response to momelotinib, with hemoglobin rising from 76 to ≥120 g/L and achieving rapid transfusion independence. This clinical benefit was substantially offset by the development of dose-limiting peripheral neuropathy. While this toxicity has been previously documented in momelotinib-treated patients, the mechanistic basis remains incompletely understood (7, 12). Potential mechanisms include direct toxic effects of JAK inhibition on peripheral sensory neurons, or alternatively, immunomodulatory effects that might exacerbate pre-existing subclinical neuropathy (10, 13).

The management approach in this case—initial dose reduction followed by therapeutic switch to ruxolitinib—demonstrates the feasibility of managing severe momelotinib toxicity while preserving hematologic benefit through alternative mechanisms. The complete resolution of neuropathy following drug cessation, coupled with sustained hemoglobin levels ≥12 g/dL on a lower ruxolitinib dose (5 mg twice daily) compared to the prior regimen (15 mg twice daily plus erythropoietin), favors discussions on several important clinical insights: *First*, the additive benefit of prior EPO exposure may have provided lasting hematologic benefit even after dose reduction and drug switching. Alternatively, the disease itself may have entered a more stable phase with decreased anemia burden. *Second*, the successful control of anemia at substantially lower ruxolitinib doses than initially required raises questions about the optimal dosing strategy in myelofibrosis-associated anemia, depending on the presence of significant splenomegaly. Current practice often employs dose escalation for suboptimal responses; however, this case demonstrates that strategic withdrawal and re-initiation with alternative agents may achieve a good efficacy-toxicity profiles. *Third*, the rapid and complete resolution of peripheral neuropathy following momelotinib cessation supports a drug-specific toxic mechanism rather than disease-related neuropathy and emphasizes the critical importance of adverse event monitoring during JAK inhibitor therapy, particularly with newer agents requiring real-world long-term safety validation. *Fourth*, chelation therapy was continued until ferritin decreased persistently below 1,000 ng/ml (October 2023). The long chelation exposure is typically associated with erythroid responses in MF, through a sustained reduction of labile iron and ROS and its beneficial actions on erythroblasts, improvement of iron-driven niche dysfunction and oxidative stress, and decreasing tissue iron, among others.

The use of JAK inhibitors represents an important therapeutic advance. However, the differential efficacy and toxicity profiles between JAKi remain incompletely characterized in the MF population. This case suggests that while momelotinib may offer exceptional anemia benefit in selected patients, the risk-benefit profile may be unfavorable, especially in patients with pre-existing neurologic vulnerability (such as prior anagrelide exposure, advanced age, or CKD). The successful retreatment with low-dose ruxolitinib supports a “step-down” dosing paradigm in selected cases rather than obligatory drug change or combination testing.

One limitation of this case is the absence of molecular follow-up, notably at the switch and re-switch times, leaving unresolved the hypothesis of a “clonal rotation” alongside the persistence of such a good clinical and biological response. Another important point is the absence of information on erythropoietin levels during the switch episodes, which might help the decision to change and the discussion of maintaining a lower dose of ruxolitinib vs. switching to momelotinib.

## Conclusion

This case documents successful management of momelotinib-induced peripheral neuropathy through therapeutic switching and dose optimization in a patient with MPL ET-secondary myelofibrosis. The favorable hematologic response to momelotinib, followed by complete resolution of neuropathy with switch to low-dose ruxolitinib and maintained disease control, illustrates the importance of individualized JAKi selection, toxicity monitoring, and flexible dose-adjustment strategies. As the armamentarium of JAKi expands, careful characterization of real-world safety profiles and development of predictive biomarkers for drug-specific toxicities will optimize patient selection and outcomes.

This case supports the continued prospective collection of adverse event data during momelotinib use and highlights the potential utility of dynamic dosing strategies and therapeutic switching in managing JAKi-related complications.

## Data Availability

The original contributions presented in the study are included in the article/supplementary material, further inquiries can be directed to the corresponding author.
